# A Complicated Parenchymal-Esophageal Fistula in Non-Small Cell Lung Cancer

**DOI:** 10.7759/cureus.22149

**Published:** 2022-02-12

**Authors:** Saba AlTarawneh, Yasmeen Obeidat, Ahmed Sherif, Yousef Shweihat, Wesam Frandah

**Affiliations:** 1 Internal Medicine, Marshall University Joan C. Edwards School of Medicine, Huntington, USA; 2 Gastroenterology, Marshall University Joan C. Edwards School of Medicine, Huntington, USA; 3 Pulmonary and Critical Care, Marshall University Joan C. Edwards School of Medicine, Huntington, USA

**Keywords:** recurrent pneumonia, self-expanding metal stents, over the scope clip, broncho-esophageal fistula, respiratory digestive fistula

## Abstract

Respiratory digestive fistula (RDF) is an abnormal communication between the airway and the digestive tract. Only 3-11% of RDF communications are parenchymal-esophageal fistulas. We present a case of a 58-year-old male who presented to the emergency department with dysphagia and cough after swallowing. He was diagnosed with stage III/B non-small cell lung cancer. The patient was previously treated with chemotherapy, radiation, and immunotherapy. Computed tomography (CT) scan of the chest with contrast showed a chronic cavitary right upper lobe lesion in the previously treated malignancy area. New right paratracheal adenopathy, right esophageal wall thickening, and bilateral lung infiltrates were also shown. Upper endoscopy with bronchoscopy and endobronchial ultrasound (EBUS) was done to evaluate mediastinal lymphadenopathy as well as dysphagia. A tract was found extending from the right lung cavity into the esophagus through the mediastinum. Esophagoscopy was subsequently performed, and a fistula was observed on the right wall of the mid-esophagus. The defect was favorable for clipping, which was successfully closed with an 11/6 traumatic over the scope clip, followed by a fully covered esophageal stent. The patient’s respiratory and gastrointestinal symptoms improved after the procedure. Follow-up barium swallow was negative for any esophageal leak. At three-month follow-up, the patient was free of recurrent pulmonary and gastrointestinal symptoms that he presented with. Palliative therapy is the targeted therapy for RDF management. RDF is either managed conservatively or with radiation, chemotherapy, or surgery to obliterate the connection. Surgical correction usually is not an option since patients typically have a poor functional status at the time of diagnosis. Considering the survival and recurrence rate medical intervention is the mainstay of treatment. Parallel dual (esophageal-bronchial) stenting has been proven to provide the best outcomes. Self-expanding metal stents (SEMS), either covered or partially covered, are used extensively to manage malignant RDF.

## Introduction

Respiratory digestive fistula (RDF) is an abnormal communication between the airway and digestive tract. Acquired malignant RDF is often secondary to esophageal, mediastinal, or pulmonary malignancies [1]. Patients with acquired RDF typically present with recurrent aspiration pneumonia, malnutrition, and respiratory failure. Aspiration pneumonia and its consequences usually lead to death rather than cancer itself [2]. The most common form of RDF is a tracheo-esophageal fistula, only 3-11% of RDF communications are attributed to peripheral communications including parenchymal-esophageal fistulas [3]. Early RDF diagnosis is crucial given the rapidly deteriorating course; it can be diagnosed with chest computed tomography (CT), endoscopy, and/or bronchoscopy [1]. We present a rare case of non-small lung cancer with recurrent aspiration pneumonia that was complicated by a tracheal-parenchymal-esophageal fistula that was managed with a single esophageal stent.

## Case presentation

**Figure 1:**
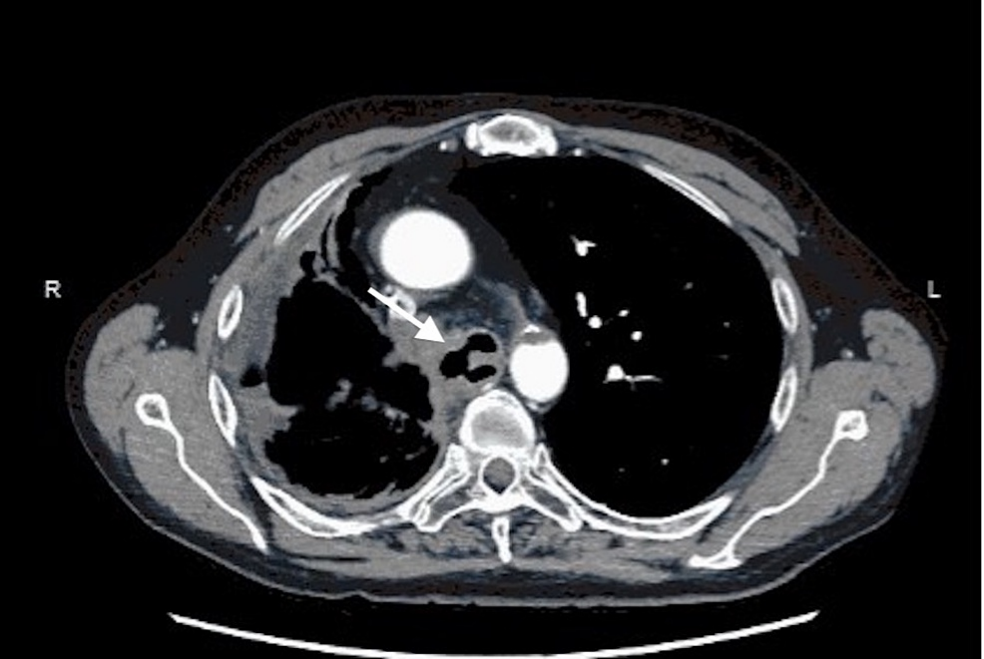
Axial computed tomography image demonstrating the broncho-parenchymal-esophageal fistula (arrow)

**Figure 2:**
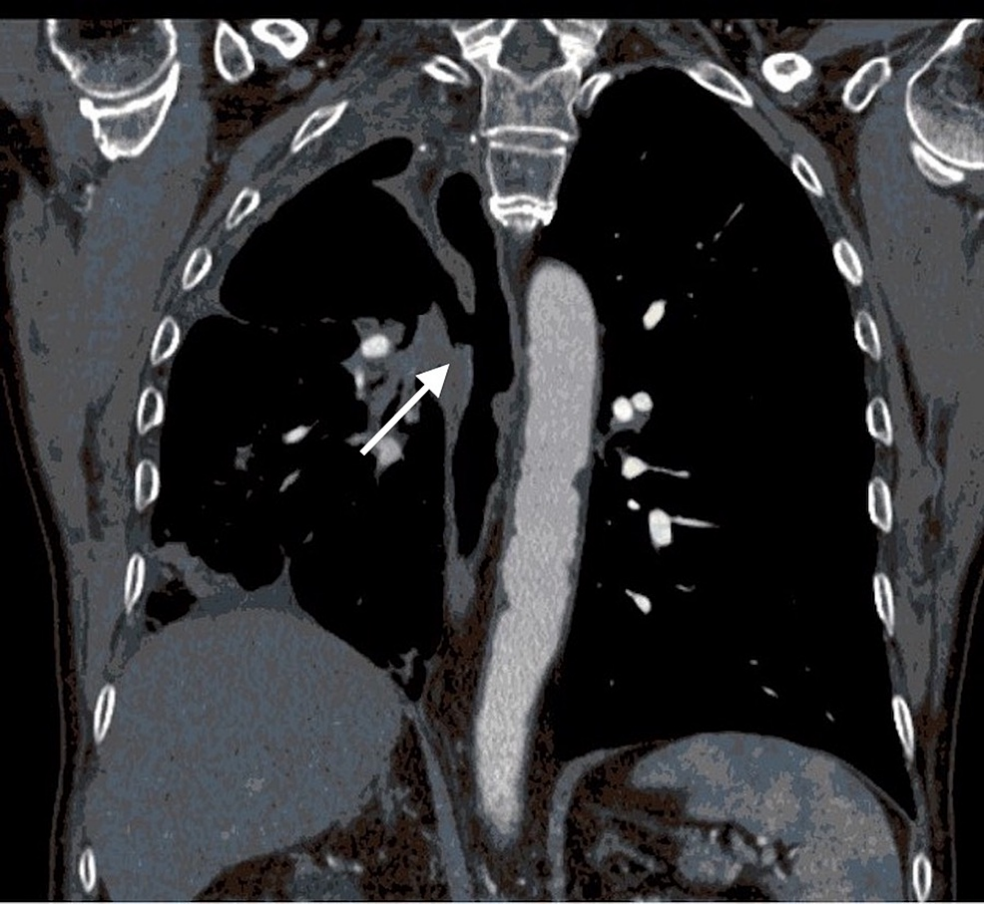
Coronal view of the computed tomography image of the parenchymal-esophageal fistula before closure (arrow)

A 58-year-old male presented to the emergency department with increasing dyspnea, dysphagia, and cough after swallowing. He was diagnosed with stage III/B non-small cell lung cancer (NSCLC) with squamous histology one year prior to presentation. The patient was previously treated with chemotherapy (carboplatin/paclitaxel), radiation, and immunotherapy (durvalumab) for one month. Past medical history included gastroesophageal reflux disease, chronic hepatitis C infection, hypertension, and a 40 pack-year smoking history. On physical examination, his vitals were unremarkable and oxygen saturation was 96% on room air. He had significant hoarseness of his voice and diminished breath sounds bilaterally with scattered rhonchi. Laboratory studies were unremarkable. Computed tomography (CT) scan of the chest with contrast showed a chronic cavitary right upper lobe lesion that replaced the previously treated malignancy, a new right paratracheal adenopathy, and esophageal wall thickening that was suggestive of parenchymal-esophageal fistula and bilateral lung infiltrates were also noted (Figures 1, 2).

**Figure 3:**
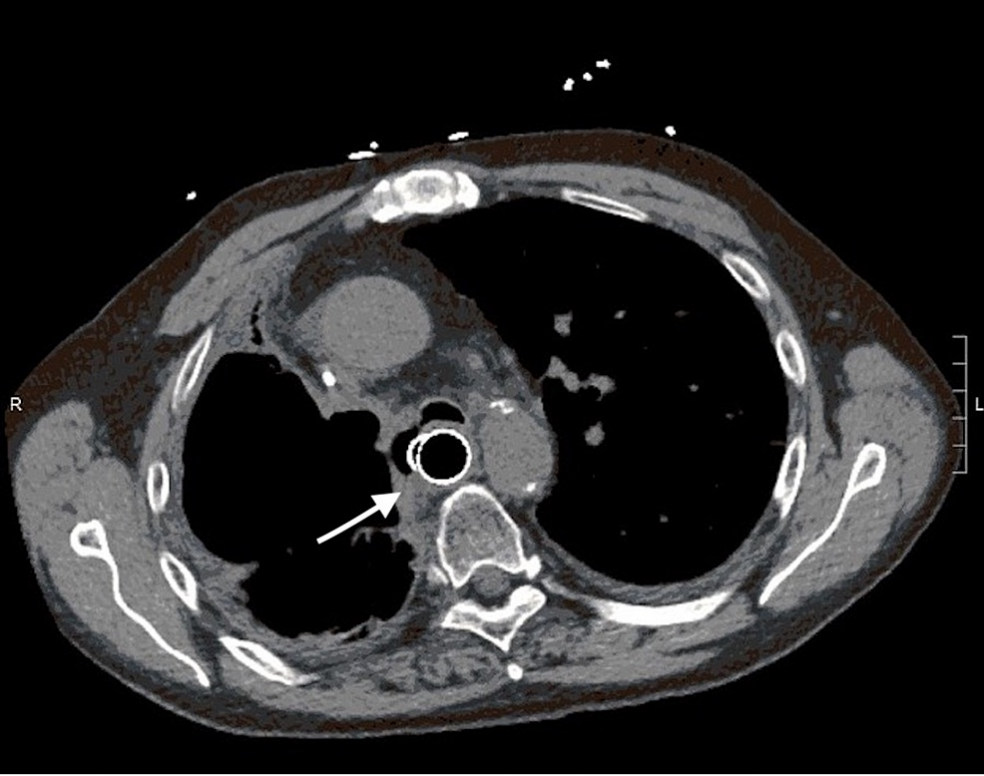
Axial computed tomography showing the parenchymal-esophageal fistula after closure (arrow)

**Figure 4:**
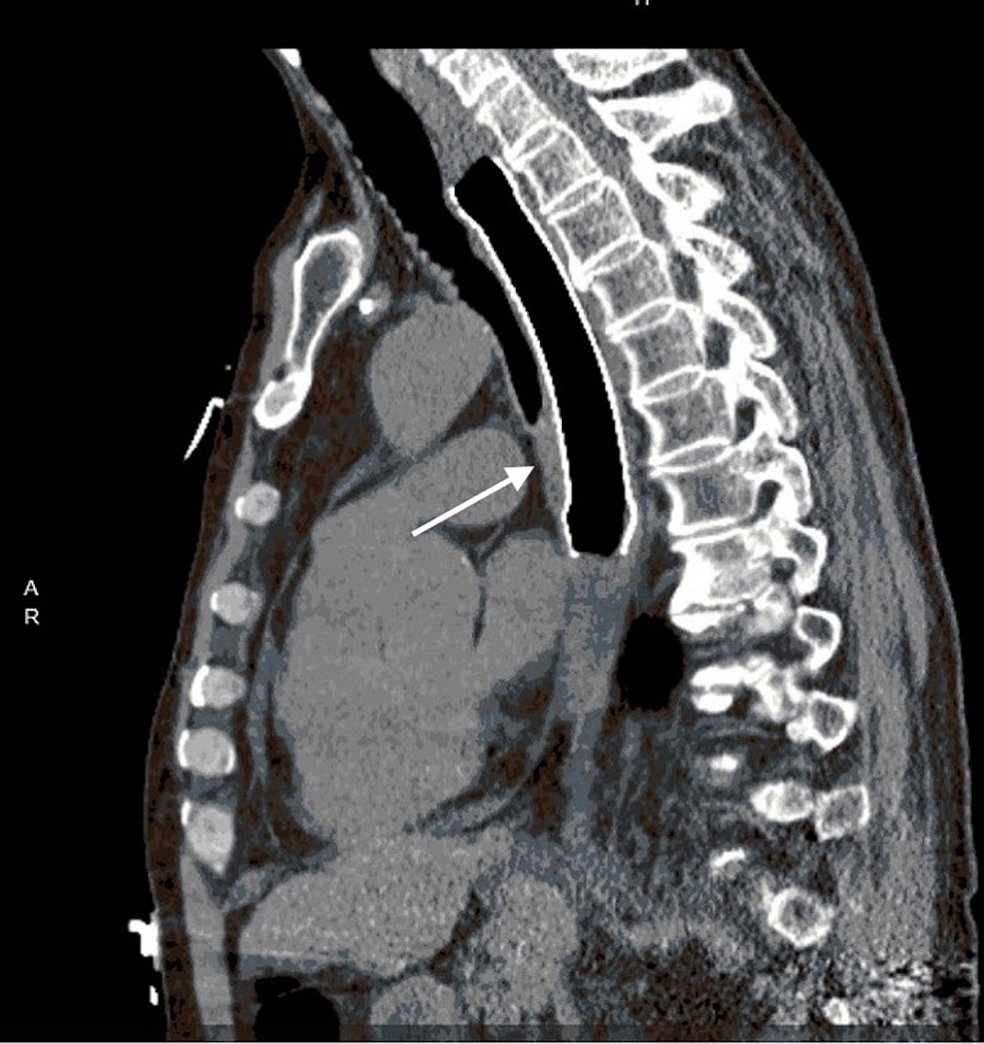
Coronal view of computed tomography image of the parenchymal-esophageal fistula after it was successfully closed by fully covered esophageal stent (arrow)

Treatment for atypical pneumonia and acute-chronic bronchitis was initiated with intravenous methylprednisolone and doxycycline, and the case was discussed in the multidisciplinary lung tumor board meeting. Based on the history of recurrent atypical pneumonia and new CT findings it was concluded that a malignancy-associated trachea-broncho-esophageal fistula should be included in his differential diagnosis. Upper endoscopy with concurrent bronchoscopy with endobronchial ultrasound (EBUS) was pursued to evaluate mediastinal lymphadenopathy as well as dysphagia. Bronchial and esophageal intervention with stent placement was a consideration. The concern was that placing only an esophageal stent might cause severe airway compromise given the lack of cartilaginous support in the adjacent bronchus. Upon bronchoscopy, the right main stem was absent and the trachea on the right opened into a huge cavity. A tract was found extending from the right lung cavity into the esophagus through the mediastinum. The bronchus intermedius was emanating out of the cavity a few millimeters lateral to the tract. Esophagoscopy was subsequently performed, and a fistula was observed on the right wall of the mid-esophagus. The defect was favorable for clipping, which was successfully closed with an 11/6 traumatic over the scope clip (OTSC), followed by a 23 mm by 10 cm fully covered esophageal stent. There was a 1.5 cm defect on the right lateral wall of the esophagus around 26 cm from the incisors in keeping with the broncho-esophageal fistula (Figures 3, 4).

The patient’s respiratory and gastrointestinal symptoms improved after the procedure. Radiographic improvement was also noted. A follow-up barium swallow was negative for any esophageal leak. At a three-month follow-up, the patient was free of recurrent pulmonary and gastrointestinal morbidity.

## Discussion

Acquired RDF is a pathologic communication between the gastrointestinal tract and airway attributed to either benign or malignant etiology. Malignancy is the most common cause for acquired RDF, esophageal cancer accounting for approximately 77%, primary lung neoplasm 16% with the remainder being due to thyroid, mediastinal, and laryngeal cancer [4]. The reported incidence of tracheoesophageal fistula (TEF) in patients with esophageal cancer is 4.5%, whereas it is 0.3% in patients with primary lung cancer [5]. Patients usually present with recurrent aspiration pneumonia and deteriorate rapidly if left unrecognized. RDF can be diagnosed initially with esophagography. CT scan can be used to determine the position of the fistula and to assess the severity. Bronchoscopy combined with esophagogastroduodenoscopy (EGD) is the most effective method to locate the fistula and perform the appropriate intervention [6]. Management of RDF is targeted toward palliative therapy. It is either managed conservatively with medication alone or with radiation, chemotherapy, or surgery to obliterate the connection between the airway and the digestive tract. Most malignant fistulas are not amenable to surgical correction since patients typically have a poor functional status at the time of diagnosis complicated by ongoing malignancy [6]. Radiation therapy has been reported to improve survival rates compared to supportive measures alone, however, radiation therapy alone is rarely used to manage RDF [7]. Chemotherapy may lead to tumor shrinkage but may lead to an increase in the fistula size [5]. Burt et al. reported that surgical intervention through fistula resection flaunted high post-procedural mortality [7]. Medical intervention, as suggested by data, is the mainstay of treatment given the survival and recurrence rate [8]. Parallel dual (esophageal-bronchial) stenting has been proven to provide the best outcomes [4]. Types of stents that have been used include mixed silicone metallic stents, silicone stents, plastic self-expanding stents, and covered or uncovered metal stents [4]. Plastic stenting is effective in palliation of dysphagia symptoms but yields a prohibitive 8-11% of acute post-procedural complications such as perforation [9]. Self-expanding metal stents (SEMS), either covered or partially covered, are used extensively to manage malignant RDF. The rate of stent migration is reported to be lower in partially covered SEMS. The ingrowth of tumors required endoscopists to repeat stenting or utilize ablation. Tumor ingrowth occurs less frequently in fully covered SEMS but is more prone to migration [9]. In our case, an esophageal stent was carefully placed with EGD, to avoid occlusion of the bronchus intermedius and right lower lobe atelectasis by placing an airway stent to cover the fistula tract on the bronchial side.

## Conclusions

Respiratory digestive fistula (RDF) is an abnormal communication between the airway and digestive tract. The most common form of RDF is a tracheoesophageal fistula, only 3-11% of RDF communications are attributed to peripheral communications including parenchymal-esophageal fistulas. Medical intervention, as suggested by data, is the mainstay of treatment given the survival and recurrence rate. Parallel dual (esophageal-bronchial) stenting has been proven to provide the best outcomes. In our case, an esophageal stent was carefully placed with EGD, to avoid occlusion of the bronchus intermedius and right lower lobe atelectasis by placing an airway stent to cover the fistula tract on the bronchial side.
